# Expression, purification and characterisation of a human anti-CDK4 single-chain variable fragment antibody

**DOI:** 10.1186/s12896-021-00729-z

**Published:** 2021-12-20

**Authors:** Jialiang Zhao, Jingjing Xu, Tianbin Yang, Xinze Yu, Cheng Cheng, Tong Zhang, Ze Ren, Na Li, Fang Yang, Guiying Li

**Affiliations:** Key Laboratory for Molecular Enzymology and Engineering of the Ministry of Education, School of Life Sciences, Jilin University, Changchun, 130012 China

**Keywords:** CDK4, Single-chain variable fragment, Soluble expression, Purification, Characterisation, Cancer cells

## Abstract

**Background:**

Cyclin-dependent kinase 4 (CDK4) when hyperactivated drives development and maintenance of most tumour types, thus prompting its use as an essential cancer treatment target and a diagnostic tool. Target-binding molecules, such as single-chain variable fragment (scFv) antibodies, hold tremendous potential for use in a wide range of cancer diagnostic and therapeutic applications.

**Results:**

A human anti-CDK4 scFv antibody (AK2) derived from a human phage display library was expressed in soluble form in *Escherichia coli* and shown to be secreted into the culture supernatant. Next, soluble AK2 within culture supernatant was successfully purified using affinity chromatography then was shown, using enzyme-linked immunosorbent assays, to bind to recombinant human CDK4 with high affinity and specificity. Further analyses of AK2 interactions with intracellular components demonstrated that AK2 recognised and interacted specifically with endogenous CDK4 and thus could be useful for detection of CDK4 within tumour cells.

**Conclusions:**

A novel anti-CDK4 scFv antibody that can recognise and interact specifically with recombinant human CDK4 and endogenous CDK4 in tumour cells was expressed and purified successfully. These results suggest that the anti-CDK4 scFv antibody may serve as a new and promising tool for achieving CDK4-targeted diagnosis, prognosis and treatment of numerous types of cancers.

**Supplementary Information:**

The online version contains supplementary material available at 10.1186/s12896-021-00729-z.

## Introduction

The timing of eukaryotic cell cycle progression is precisely controlled by a regulatory network that primarily involves activities and specificities of cyclin-dependent kinases, including CDK4 (cyclin-dependent kinase 4), which plays a key role in cell cycle initiation [1]. More specifically, CDK4 forms a complex with D-type cyclin that acts as a kinase by phosphorylating Rb protein, an event that induces E2F transcription factor release that triggers cell entry into G1/S phase transition [2]. Numerous studies have demonstrated that abnormal CDK4 levels or activation states are closely related to tumourigenesis and progression of various cancers [3]. Notably, amplification or rearrangements of the CDK4-encoding gene have been shown to cause overexpression of CDK4 protein that has almost universally been associated with many types of solid tumours and hematologic malignancies [3–5]. In addition, CDK4 inhibitor proteins, including p16^INK4a^ (CDKN2A), are often inactivated in many cancers [6]. Such alterations of CDK4 gene expression can result in excessive activation of CDK4 kinase within tumour cells that, in turn, drives cell proliferative disorders that can trigger tumourigenesis of most tumour types [7]. Genetic testing efforts have revealed that expression of individual cyclins and CDKs is most likely dispensable for the proliferation of cells within healthy tissues [8]. By contrast, changes in cyclin and CDK genes that alter expression of these proteins have increasingly been implicated in certain types of cancers, with each specific cancer type shown to be associated with a specific type of genetic alteration [9]. Moreover, an observed association of cyclin D-CDK4/6 activity with tumour maintenance has been noted in several previous studies. Based on these results, research efforts are currently being directed toward the development of anti-cancer treatment strategies based on utilisation of CDK4 as a target [10]. In fact, three small inhibitors targeting CDK4 have already been approved for clinical treatment of ER-positive breast cancer [11–13]. However, application and development of anti-tumour treatments targeting CDK4 still require more extensive and in-depth studies. For instance, new strategies are needed to solve the problem of emerging drug resistance and side effects associated with use of small molecule-based anti-cancer drugs [14–17].

Antibody-based treatments for numerous disorders constitute the fastest-growing class of drugs on the market, due to their excellent stability, specificity and adaptability [18]. Antibody-based drugs include single-chain variable fragments (scFvs), which are comprised of the light chain variable region and the heavy chain variable region of an antibody connected by a linker peptide. Each scFv represents the most minimal active component of its corresponding full-length human antibody by providing comparable antigen binding affinity and specificity. However, scFvs possess several advantages over their full-length counterparts, such as small size, strong penetrability, weak immunogenicity and ease of modification [19–21]. In addition, scFvs offer advantages of superior specificity and fewer side effects as compared to other types of small molecule-based drugs [22]. Given the abovementioned advantages, scFvs are currently in widespread use for treating and diagnosing various diseases, including cancer [23]. For example, PD-1-blocking scFv-secreting chimeric antigen receptor (CAR) T cells have been shown to possess paracrine and autocrine activities that improve anti-tumourigenic activities of CAR-T cells and bystander T cells against syngeneic and xenogeneic mouse models of human clinical PD-L1^+^ hematologic and solid tumours [24]. In addition to cancers, scFvs have been used for treatment of numerous diverse types of disorders, including anti-tau and anti-Aβ scFvs, which have been shown to exert significant beneficial effects as treatments for neurodegenerative diseases [25]. In another study, scFvs were used to treat dextran sulphate sodium-induced colitis in mice, whereby introduction of bacteria expressing anti-TNF scFv into mice via gavage led to mucosal delivery of the scFv and subsequent alleviation of colitis [26]. In other work evaluating an scFv for prevention of acute acetaminophen-induced liver injury, administration of an scFv against PHD2 (prolyl hydroxylase 2) prevented liver injury by partially improving angiogenesis and maintaining redox homeostasis [27, 28]. Meanwhile, other scFvs have been developed to detect biomarkers associated with numerous diseases [29–31]. A human anti-cyclin D1 scFv with high antigen affinity and specificity which was recently obtained via screening of phage display antibody libraries, was shown to induce an excellent anti-tumour effect by inhibiting activation of CDK4 present within cells [32]. In summary, designing and construction of human anti-CDK4 scFvs will likely facilitate future studies to understand CDK4 functions in normal and tumour cells, while also leading to development of key anti-cancer drugs that target CDK4.

In this study, a human anti-CDK4 scFv antibody, named AK2, expressed and secreted in soluble form by *Escherichia coli* (*E. coli*) HB2151 cells was obtained by screening a semi-synthetic human scFv phage library. Purification of AK2 was easily performed using affinity chromatography then purified AK2 was confirmed and verified to be specific for CDK4 using enzyme-linked immunosorbent assays (ELISAs). High-affinity binding and specificity of AK2 for recombinant human CDK4 (rhCDK4) were verified using competitive ELISAs, while a non-competitive ELISA was employed to measure AK2 binding affinity to CDK4. Moreover, AK2 was confirmed to interact with intracellular CDK4 using immunofluorescence, western blot and co-immunoprecipitation experiments. The results of this study provide evidence that a new promising anti-tumour treatment strategy, anti-CDK4 scFv, may be useful for tumour diagnosis and CDK4-targeting therapeutic applications.

## Results

### Soluble AK2 in culture supernatant specifically binds to CDK4

**Fig. 1:**
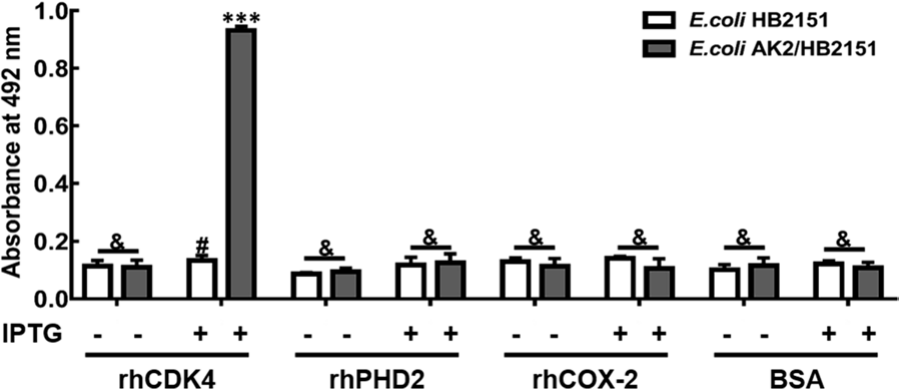
CDK4-binding activity of soluble AK2 in *E. coli* HB2151 culture supernatant. Coating of microtiter plates was conducted using equal concentrations of rhCDK4 or irrelevant antigens (rhPHD2, rhCOX-2, or BSA). Next, plates were incubated sequentially with culture supernatants of either IPTG-induced or uninduced *E. coli* AK2/HB2151 cells followed by incubation with anti-V5 tag monoclonal antibody then HRP-conjugated anti-mouse IgG. The group incubated with supernatant of *E. coli* HB2151 served as the negative control (NC) group. (*** *P* < 0.001 vs. corresponding irrelevant proteins controls or NC group, # *P* > 0.05 vs. corresponding irrelevant proteins controls, & represents *P* > 0.05). Abbreviations: rhCDK, recombinant human cyclin-dependent kinase; rhPHD, recombinant human prolyl hydroxylase; rhCOX, recombinant human cyclooxygenase; BSA, bovine serum albumin; IPTG, isopropyl β-D-1- thiogalactopyranoside

Phagemid DNA encoding AK2 was obtained by screening a phage library [33] for phages binding to rhCDK4 and the deduced amino acid sequences of AK2-VL and AK2-VH were shown in Additional file 1: Fig S1. Then the AK2 phagemid was used to infect amber non-suppressive *E. coli* HB2151 cells to obtain *E. coli* clone AK2/HB2151. Next, addition of IPTG to an *E. coli* AK2/HB2151 culture led to expression and secretion of soluble AK2 into the culture supernatant, since the cloned DNA of AK2/HB2151 carries a pelB leader peptide sequence upstream of AK2 and a succinic acid stop codon between the *AK2* gene and the *gIII* gene. To verify production of secreted AK2 and determine its binding activity, the supernatant of an *E. coli* AK2/HB2151 culture was collected after overnight IPTG induction of AK2 expression and then was added to microtiter plates that had been pre-coated with rhCDK4 or an irrelevant protein to conduct a non-competitive ELISA. As shown in Fig. 1, absorbance measurements at 492 nm indicated that binding of soluble AK2 within the abovementioned culture supernatant to rhCDK4-coated wells was significantly greater (*P* < 0.001) than binding to wells coated with irrelevant protein. However, binding of the abovementioned culture supernatant without AK2 to rhCDK4-coated wells was similar to binding to wells coated with irrelevant proteins (*P* > 0.05). Moreover, no significant differences were found between results for the negative control (NC) group to rhCDK4-coated wells and to wells coated with irrelevant proteins (*P* > 0.05). Thus, AK2 was successfully expressed in soluble form by *E. coli* AK2/HB2151 cells and was secreted into the culture supernatant. In addition, soluble AK2 was confirmed to specifically bind to rhCDK4.

### Purification and identification of AK2

**Fig. 2:**
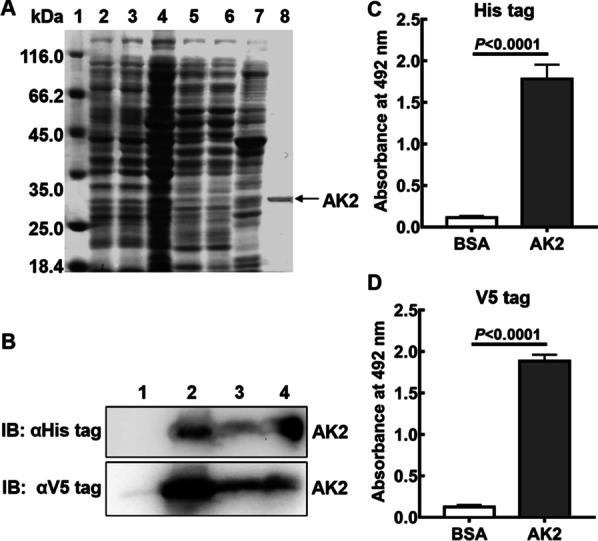
Purification and identification of AK2. **a** Analysis of expression and purification of AK2 using 12% SDS-PAGE. Lane 1: Protein molecular weight standard; Lane 2: Lysate of *E. coli* AK2/HB2151 not induced with IPTG; Lane 3: Lysate of *E.coli* AK2/HB2151 induced by IPTG; Lane 4: Crude proteins adsorbed to Ni^2+^-NTA column; Lane 5: Flowthroughs of crude protein sample from Ni^2+^-NTA column; Lane 6: Washes from Ni^2+^-NTA column with binding buffer; Lane 7: Washes from Ni^2+^-NTA column with washing buffer; Lane 8: Elution from Ni^2+^-NTA column with elution buffer. **b** Western blot analysis of purified AK2 using anti-V5 tag monoclonal antibody and anti-His tag monoclonal antibody probes, respectively. Lanes 1 and 2: Cell lysate of *E. coli* AK2/HB2151 induced without or with IPTG, respectively; Lane 3: Culture supernatant of *E. coli* AK2/HB2151 induced by IPTG; Lane 4: Purified AK2 protein. The original blot image is shown in Additional file 1: Fig S2. **c**, **d** Recognition of purified AK2 by ELISA. Purified AK2 was coated onto the microtiter plates to conduct ELISA using anti-His tag monoclonal antibody (**c**) and anti-V5 tag monoclonal antibody (**d**). Wells coated with BSA served as the negative control (NC)

To purify soluble AK2, crude protein was first isolated from *E. coli* AK2/HB2151 cell culture supernatant using an ammonium sulphate precipitation method that had been optimised in a previous pilot experiment. The results of that work had demonstrated that 50% ammonium sulphate saturation was optimal for precipitating crude protein from culture medium-based supernatant by minimising contamination with other proteins and maximising AK2 yield, thus facilitating subsequent purification steps. Using this method, AK2 within the crude protein preparation was enriched then was further purified using a Ni^2+^-NTA affinity column. For affinity chromatography, binding buffer containing 10 mM imidazole was used to promote binding of most of the AK2 to the Ni^2+^-NTA column. After AK2 bound to the column, wash buffer containing 40 mM imidazole was applied to the column to remove non-specifically adsorbed proteins then elution buffer containing 500 mM imidazole was used to elute purified AK2 from the column. As shown in Fig. 2a, SDS-PAGE analysis of the eluted fraction indicated that the eluant primarily contained a protein that appeared in the gel as a single band of molecular weight of approximately 30 kD.

Due to the fact that AK2 contains a 6 × His tag and a V5 tag at its C-terminus, detection of AK2 was conducted using western blots probed with anti-His tag or anti-V5 tag antibodies. Western blot results revealed that cell lysates and culture supernatant of IPTG-induced *E. coli* AK2/HB2151 cells and purified AK2 protein contained a clear protein band with a molecular weight of 30 kD, while no protein bands were observed in cell lysates of *E. coli* AK2/HB2151 without IPTG induction (Fig. 2b). These results thus indicated that AK2 was successfully expressed in *E. coli* AK2/HB2151 cells and secreted into the culture supernatant, while also confirming that soluble AK2 had been successfully purified. Further confirmation of successful AK2 purification was obtained using ELISAs incorporating anti-His tag or anti-V5 tag AK2 detection antibodies (Figs. 2c, d), which generated markedly higher absorbance values at 492 nm for microtiter plates coated with AK2 than absorbance values obtained for NC wells coated with BSA (*P* < 0.0001). Therefore, these results confirmed successful purification of AK2 from a culture supernatant of IPTG-induced *E. coli* AK2/HB2151 cells.

### Purified AK2 bound specifically to rhCDK4

**Fig. 3:**
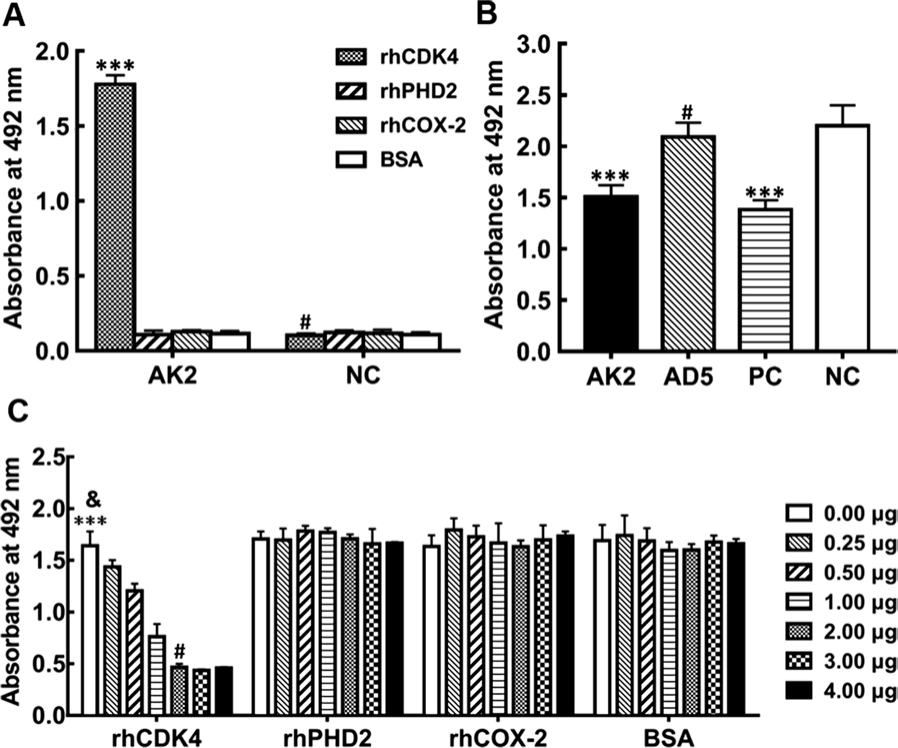
Purified AK2 specifically bound to rhCDK4. **a** CDK4-binding activity of purified AK2. Microtiter plates were coated with rhCDK4 or irrelevant proteins (rhPHD2, rhCOX-2 or BSA) then wells were incubated sequentially with purified AK2, anti-V5 tag antibody then HRP-conjugated IgG to conduct the ELISA assay. Wells lacking purified AK2 served as the negative control (NC) (*** *P* < 0.001 vs. corresponding irrelevant protein controls or NC group, # *P* > 0.05 vs. corresponding irrelevant protein controls). **b** AK2 competition with anti-CDK4 polyclonal antibody for CDK4 binding. Wells coated with rhCDK4 were incubated with AK2 that had been pre-incubated with irrelevant scFv AD5, anti-CDK4 mouse monoclonal antibody (PC group) or BSA (NC group) in order to determine whether AK2 could competitively bind to CDK4 in the presence of anti-CDK4 rabbit polyclonal antibody. The ELISA assay was then conducted by sequential probing with anti-CDK4 rabbit polyclonal antibody then HRP-conjugated goat anti-rabbit IgG followed by the addition of HRP chromogenic solution (*** *P* < 0.001 vs. corresponding pre-incubation with irrelevant scFv AD5 or NC group, # *P* > 0.05 vs. NC group). **c** Specificity of binding of AK2 to CDK4. Purified AK2 that had been pre-incubated with different concentrations of CDK4 or with other irrelevant proteins was added to wells coated with rhCDK4 in order to assess specificity of binding of AK2 to CDK4. The absorbance at 492 nm was measured after sequential addition of anti-V5 Tag monoclonal antibody, HRP-conjugated goat anti-mouse IgG then HRP chromogenic solution (*** *P* < 0.001 vs. corresponding pre-incubation with different concentrations of CDK4, # *P* > 0.05 vs. corresponding pre-incubation with 3.00 μg and 4.00 μg of CDK4, & *P* > 0.05 vs. corresponding pre-incubation with other irrelevant proteins). Abbreviations: rhCDK, recombinant human cyclin-dependent kinase; rhPHD, recombinant human prolyl hydroxylase; rhCOX, recombinant human cyclooxygenase; BSA, bovine serum albumin; IPTG, isopropyl β-D-1- thiogalactopyranoside

The binding activity of purified AK2 to rhCDK4 was first verified via non-competitive ELISA. Coated wells were incubated sequentially with purified AK2 (or with PBST with 3% non-fat milk as NC) then with anti-V5 tag monoclonal antibody. Next, HRP-conjugated goat anti-mouse IgG was added to wells then OPD (o-phenylenediamine) was added and absorbance measurements were taken at 492 nm. As shown in Fig. 3a, absorbance values were significantly higher for rhCDK4-coated wells incubated with purified AK2 than for wells coated with irrelevant protein (rhPHD2, rhCOX-2, or BSA) and NC wells (*P* < 0.001). Moreover, no significant differences were found between results for the irrelevant protein groups and NC groups, thus demonstrating that purified AK2 bound specifically to rhCDK4.

To further verify the specificity of AK2 binding to CDK4, first we conducted competitive ELISAs that demonstrated that AK2 could compete with rabbit anti-CDK4 antibody for binding to rhCDK4. As shown in Fig. 3b, when rhCDK4 was incubated only with rabbit anti-CDK4 antibody (the NC group), the absorbance at 492 nm was 2.20 ± 0.20. In the positive control group, when rhCDK4 was sequentially incubated with mouse anti-CDK4 monoclonal antibody then with rabbit anti-CDK4 antibody, the absorbance at 492 nm was 1.38 ± 0.08, for an inhibition rate of approximately 37%. Following sequential incubation of rhCDK4 with AK2 then with rabbit anti-CDK4 antibody, the absorbance at 492 nm was noticeably reduced to 1.51 ± 0.09. However, when rhCDK4 was sequentially incubated with the irrelevant scFv (AD5) [34] then with rabbit anti-CDK4 antibody, the absorbance at 492 nm was 2.09 ± 0.12, which was not significantly different from that of the NC group (*P* > 0.05). According to these results, AK2 pre-incubation significantly prevented binding from occurring between the rabbit anti-CDK4 antibody and CDK4 (*P* < 0.001), achieving an inhibition rate of approximately 31%.

We then conducted competitive ELISA assays, which also showed that pre-incubation of AK2 with rhCDK4 markedly blocked binding of AK2 to CDK4 (Fig. 3c). In this experiment, AK2 that had been pre-incubated with rhCDK4 or irrelevant proteins was added to rhCDK4-coated wells followed by assessment of competitive binding using anti-V5 tag monoclonal antibody. When purified AK2 was pre-incubated with different concentrations of rhCDK4, binding activity of AK2 to wells coated with rhCDK4 (as detected using anti-V5 tag antibody) was significantly lower than AK2 binding to NC wells containing only AK2 in PBST with 3% non-fat milk (*P* < 0.001). By contrast, pre-incubation of AK2 with different concentrations of irrelevant proteins (instead of rhCDK4) resulted in no suppression of AK2 binding to rhCDK4-coated wells (Fig. 3c). When the amount of rhCDK4 in 100 μL of the pre-incubation mixture was increased to 2.00 μg, the inhibition rate reached its highest level of almost 80%. Taken together, all of these results indicate that purified AK2 specifically bound to rhCDK4.

**Fig. 4:**
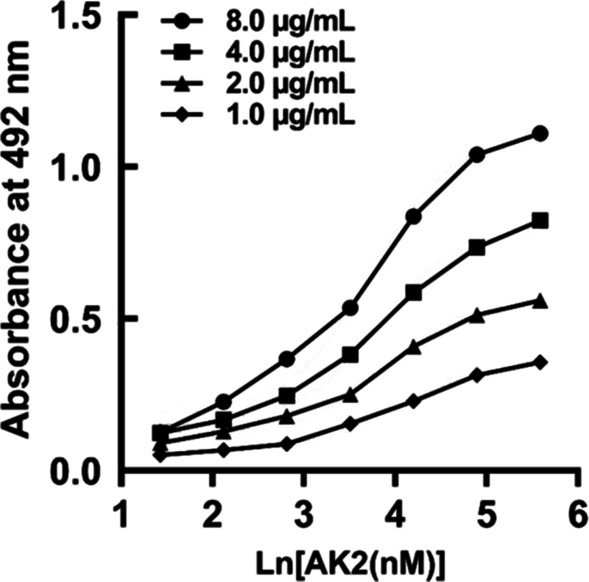
Determination of affinity of AK2 by noncompetitive ELISA. Microtiter plates coated with 8.0, 4.0, 2.0 or 1.0 μg/mL of rhCDK4 were incubated with serial dilutions of AK2 as part of the non-competitive ELISA assay. Affinity constants were calculated based on methods as described in the “Materials and Methods” section

To determine the affinity constant of AK2 binding to rhCDK4, we used the method described in previous research [32, 34, 35]. Using four concentrations of rhCDK4 (8.0, 4.0, 2.0 and 1.0 μg/mL) coated onto wells of microtiter plates, four sigmoid curves of ODs vs. logarithms of AK2 concentrations coated onto wells (Fig. 4) were generated, with results shown in Table 1. Based on the four curves and the formula used to calculate the affinity constant, we obtained six affinity constants (3 for n = 2, 2 for n = 4 and 1 for n = 8) for AK2. The average affinity constant (*K*_aff_) of AK2 binding to CDK4 was approximately 2.48 × 10^7^ M^−1^ (Table 1).

**Table 1:** The affinity of AK2 with CDK4 according to the non‐competitive ELISA assay

Antibody	Affinity [× 10^7^ M^−1^]			
	*K*_n=2_	*K*_n=4_	*K*_n=8_	Mean ± SD
AK2	2.77, 2.28, 2.37	2.58, 2.31	2.55	2.48 ± 0.17

*K*_n=2_ is the collection of affinity calculated from every 2 curves differed in amount of coated CDK4 by twofold dilution. Analogized from this, *K*_n=4_ and *K*_n=8_ were calculated and listed above

### AK2 bound to endogenous CDK4 in tumour cells

**Fig. 5:**
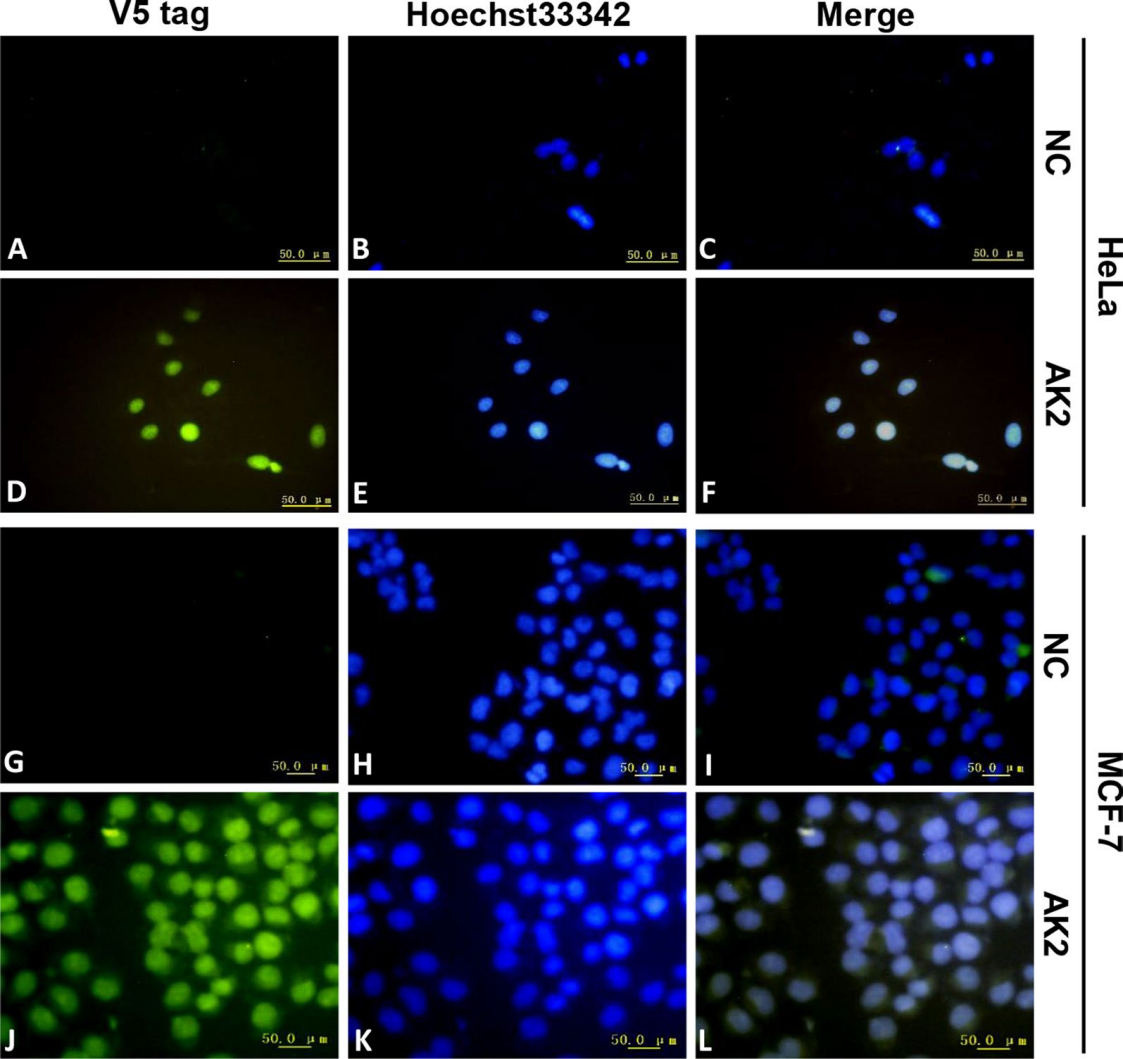
Binding activity of AK2 to CDK4 in cells as measured via an immunofluorescence assay. Cells were incubated sequentially with purified AK2, anti-V5 tag antibody then FITC-conjugated IgG as part of the immunofluorescence assay, with Hoechst33342 used to stain nucleus. Cells not incubated with purified AK2 served as the negative control (NC). A-F: HeLa cells; G-L: MCF-7 cells

After we determined that AK2 could bind specifically and with high affinity to rhCDK4, we next tested whether AK2 could also bind to endogenous CDK4 within tumour cells. First, immunofluorescence analysis was performed using HeLa and MCF-7 tumour cell lines stained with Hoechst 33,342, a fluorescent dye often used to stain cell nuclei that emits blue fluorescence under ultraviolet irradiation. HeLa cells and MCF-7 cells were incubated sequentially with purified AK2 and anti-V5 tag antibody followed by incubation with FITC-labelled goat anti-mouse IgG. Since CDK4 mainly accumulates and functions within the cell nucleus, green fluorescence would be observed if AK2 bound to endogenous CDK4. As shown in Fig. 5, HeLa and MCF-7 cells incubated with AK2 were observed to emit clear green fluorescence that was localised to the nucleus based on merging of data with Hoechst staining results. However, barely any green fluorescence was observed for NC group cells not incubated with AK2, thus indicating that AK2 specifically bound to CDK4 within the nucleus.

**Fig. 6:**
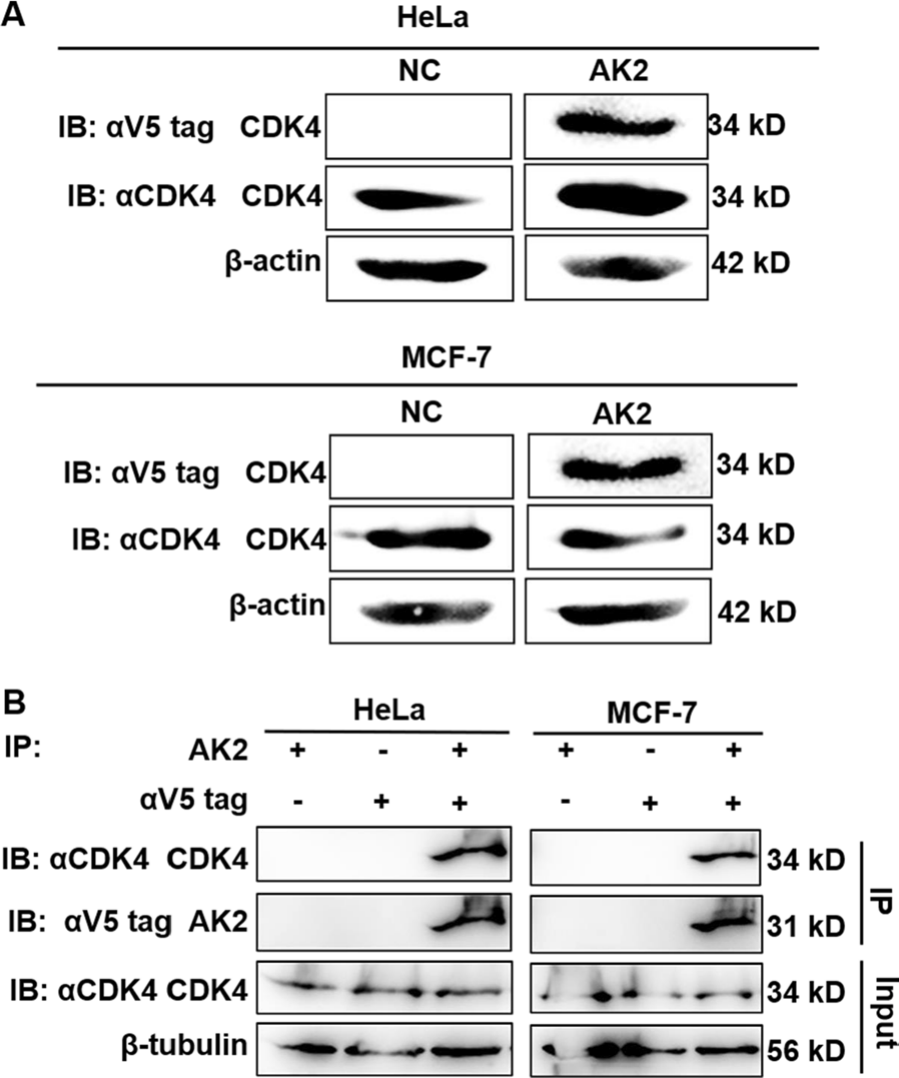
AK2 bound to endogenous CDK4 within cells. **a** Detection of binding between AK2 and CDK4 within cells as shown using western blot analysis. Proteins of HeLa and MCF-7 cells were separated using 12% SDS-PAGE then were transferred to PVDF membranes. PVDF membranes were sequentially probed with AK2 and anti-V5 tag antibody to detect CDK4, while PVDF membranes probed only with anti-V5 tag antibody served as the NC, while β-actin served as loading control. The original blot image is shown in Additional file 1: Fig S3. **b** Analysis of interactions between AK2 and CDK4 in cells using co-immunoprecipitation assays. HeLa and MCF-7 cell lysates pre-incubated with AK2 were co-immunoprecipitated with anti-V5 tag antibody then were subjected to western blot analysis to detect CDK4 binding. Lysates without added purified AK2 served as the negative control (NC). Mouse IgG served as NC for probing with anti-V5 tag antibody. Western blots of HeLa and MCF-7 cell lysates served as input control, while β-tubulin served as loading control. The original blot image is shown in Additional file 1: Fig S4

We next used western blot analysis to determine whether AK2 bound to endogenous CDK4 present within cell lysates of tumour cell lines HeLa and MCF-7 by conducting SDS-PAGE followed by transfer of isolated proteins to PVDF membranes. As shown in Fig. 6a, when PVDF (polyvinylidene difluoride) membranes were incubated with either commercial anti-CDK4 or anti-β-actin antibody, a specific 34-kD band for CDK4 was detected and a specific 42-kD band for β-actin was detected. Western blot analysis revealed obvious bands with the same molecular weight as that of CDK4 that were only observed on PVDF membranes incubated with AK2, while blots of the NC group that were not incubated with AK2 exhibited no bands, indicating that AK2 recognised and bound to endogenous CDK4 within tumour cells.

Next, co-immunoprecipitation (co-IP) experiments, which provide a direct and effective means for detecting protein–protein interactions, were used to assess interactions between AK2 or anti-V5 antibody and CDK4 present within cells. First, co-IP was conducted of lysates of HeLa and MCF-7 cells after treatment of cells with AK2 then AK2-CDK4 complexes were detected using an anti-CDK4 antibody. As shown in Fig. 6b, western blot results of input proteins showed that the total protein input of each group was the same and contained the same amount of CDK4. However, CDK4-specific 34-kD bands only appeared after lysates were subjected to co-IP with AK2 and the anti-V5 tag antibody, while no bands appeared in IgG and NC groups without AK2. Taken together, these results further demonstrated that AK2 bound specifically to endogenous CDK4.

## Discussion

CDK4, a key factor associated with cell cycle regulation, is currently being utilised as an important target for cancer treatment [8]. In fact, CDK4/6 specific small molecule inhibitors already have been approved by the FDA for use in treating breast cancer [8, 11–13]. However, successful use of small molecule inhibitors has been limited by drug resistance [14, 16] and side effects [15, 17], prompting researchers to develop novel CDK4 targeting methods to better understand CDK4 function toward development of treatment strategies based on CDK4 inhibition. In addition to small molecule inhibitors, commonly used strategies for inhibiting intracellular target proteins include gene knockdown techniques on the nucleotide level such as siRNA and intracellular antibody (intrabody) techniques on the protein level such as scFv-based intrabody [21]. ScFv-based intracellular antibodies, are genetically engineered scFv antibodies produced intracellularly that bind to antigens expressed within the same living cell [36]. Increasing evidence has demonstrated that scFv-based intrabody technology provides an interesting alternate strategy for achieving specific knockout protein phenotypes of intracellular target molecules that bypasses various limitations and disadvantages of other knockdown techniques [21, 37]. Therefore, an anti-CDK4 scFv that specifically recognizes endogenous CDK4 in tumor cells will provide a new and promising tool for achieving CDK4-targeted diagnosis and treatment of numerous types of cancers. Toward these goals, in our previous study we constructed a prokaryotic expression vector expressing rhCDK4 then purified the rhCDK4 protein [38]. In the current work, the phagemid DNA encoding a human scFv antibody against CDK4 (AK2) was obtained by screening a human semi-synthetic scFv phage library [33] using rhCDK4 as the antigen. AK2 in soluble form was expressed and prepared using *E. coli* HB2151 cells. We then showed that AK2 specifically targeted the rhCDK4 protein with high affinity and specificity. Moreover, AK2 specifically recognised and interacted with endogenous CDK4 within tumour cells, thus demonstrating that we successfully obtained a specific human scFv for CDK4.

Expression of soluble antibody proteins is the first required step for determining antibody protein quality and functional activity. In fact, a large number of studies have been focused on improving expression of soluble and stable scFv antibodies using various bacterial and yeast expression systems [20, 39–41]. Among existing expression systems, those based on *E. coli* have become popular for use in scFvs preparation, although use of an *E. coli*-based system to generate the two intrachain disulfide bonds necessary for antibody folding and antigen-binding activity can be challenging [20]. To address this problem, researchers have designed various strategies to produce functional scFvs by promoting their accumulation within the periplasm, where the oxidising environment supports disulfide bond formation and corrects scFv folding [20, 37, 42–45]. In this work, AK2 was expressed in soluble form in *E. coli* HB2151 cells after it was isolated from the scFv phage library. The prokaryotic expression vector used in this experiment contains a pelB leader peptide sequence that supports scFv accumulation within the bacterial periplasm before the scFv is secreted by the cell into the extracellular milieu. The oxidising environment of the periplasmic cavity provides a beneficial environment that supports formation of a single-chain antibody in its native conformation, while secretion of the antibody by cells facilitates later purification of the target antibody while minimising scFv cleavage by bacterial proteases. Here, ELISA results demonstrated that both AK2-containing culture supernatant of IPTG-induced cells and purified AK2 possessed significantly higher binding activities to rhCDK4 as compared to the corresponding NC, thus indicating that AK2 was successfully expressed in soluble form by *E. coli* AK2/HB2151 cells and was secreted into the culture supernatant. In addition, the current results show that AK2 can specifically bind to rhCDK4 and endogenous CDK4 in tumour cells, which indicates that AK2, as a single-chain antibody, maintains its unique biological structure and biological activity in this soluble expression and purification system.

The application of the antibody in the follow-up needs to ensure that it has high specificity and high affinity. The specificity of the binding between the purified AK2 and rhCDK4 was proved by non-competitive ELISA and competitive ELISA with irrelevant proteins. The results showed that irrelevant proteins cannot be recognised by AK2 and have no ability to compete with CDK4 for binding to AK2 while the pre-incubation with rhCDK4 inhibited almost 80% of binding of AK2 to rhCDK4. Then, competitive ELISA of commercial antibodies and affinity determination results showed that AK2 targeted the rhCDK4 protein with high affinity enough to compete with commercial antibodies, with an affinity constant of about 2.48 × 10^7^ M^−1^. Due to the difference between the rhCDK4 and the endogenous CDK4 caused by the different expression environment, further test was conducted to determine the specific binding activity of AK2 with intracellular CDK4. In this study, using purified AK2 protein as antibody, we successfully analysed CDK4 in cells using western blotting and coimmunoprecipitation assays that together indicated that AK2 protein could recognise and interact specifically with endogenous CDK4 within tumour cells and thus could be useful for detection of CDK4 within tumour cells.

Recently, intrabody techniques have been used by us to generate several scFvs that can specifically block functions of key molecules within cells when acting as anti-tumour treatments or providing protection against liver injury [28, 32]. Based on the above results and our previous work, we will be able to generate intracellular AK2 in the future and achieve the specifically phenotypic knockdown of intracellular CDK4. In view of this, AK2 has great potential for use in clinical diagnosis and prognosis of tumours based on monitoring of CDK4 biomarkers, while also holding promise as an intrabody-based anti-tumour therapy that acts by targeting intracellular CDK4.

## Conclusion

We expressed and purified novel anti-CDK4 scFv antibody AK2 to target CDK4 as an important anti-cancer therapeutic strategy. Purified AK2 bound to rhCDK4 with high specificity and affinity and recognised and interacted specifically with endogenous CDK4 within tumour cells. These experimental results lay the foundation for subsequent studies based on internalisation of scFv antibodies in order to elucidate the function of CDK4 and target CDK4 within tumour cells. The results of this study demonstrate that use of an anti-CDK4 scFv antibody is an exciting and promising strategy for achieving CDK4-targeted cancer diagnosis, prognosis and treatment.

## Materials and methods

### Materials

The phagemid AK2 encoding human anti-CDK4 scFv fused with V5 tag was obtained by screening a human semi-synthetic scFv phage library, which along with *Escherichia coli* (*E. coli*) HB2151 cells were generously provided by professor Yan Wang, Navy General Hospital, Beijing, China [33]. Anti-CDK4 rabbit polyclonal antibody (sc-601) and mouse monoclonal antibody (sc-23896) were purchased from Santa Cruz Biotechnology (Santa Cruz, CA, USA). Anti-β-tubulin mouse antibody (EM0103), antibodies against the His tag (M0812-3) and V5 tag (M1008-2) were purchased from Huabio (Hangzhou, China). Anti-β-actin rabbit antibody (20,536-1-AP), Mouse IgG and horseradish peroxidase (HRP)-conjugated goat anti-rabbit IgG (SA00001-2) and goat anti-mouse IgG (SA00001-1) were purchased from the Proteintech Group, Inc (Chicago, IL, USA). Ni^2+^-NTA columns were purchased from QIAGEN (Hilden, Germany). PD-10 desalting columns were purchased from GE Healthcare Bio-Sciences AB (Uppsala, Sweden).

### Expression and purification of soluble AK2

The phagemid AK2 was employed to infect *E. coli* HB2151 cells to generate *E. coli* AK2/HB2151, which can express soluble AK2. Next, expression of soluble AK2 protein by *E. coli* AK2/HB2151 cells was induced by addition of 1 mM IPTG (isopropyl β-D-1-thiogalactopyranoside) followed by incubation overnight with shaking at 30 °C to induce expression and secretion of soluble AK2. Next, the culture supernatant of *E. coli* AK2/HB2151 cells was collected by centrifugation then soluble proteins within the centrifuged supernatant were precipitated by adding ammonium sulphate followed by another centrifugation step. The pellet containing precipitated AK2 was dissolved in ice-cold PBS then was dialysed against PBS buffer overnight at 4 °C. After centrifugation of the dialysed AK2-containing preparation, the clear supernatant was collected and subjected to affinity chromatography using a Ni^2+^-NTA column equilibrated with binding buffer (50 mM phosphate buffer, pH 7.4, 500 mM NaCl, 10 mM imidazole). After applying the supernatant to the column in binding buffer followed by washing of the column with wash buffer (50 mM phosphate buffer, pH 7.4, 500 mM NaCl, 40 mM imidazole), the column was eluted with elution buffer (50 mM phosphate buffer, pH 7.4, 500 mM NaCl, 500 mM imidazole). Flowthroughs from the column during the elution step were collected and AK2 purification was verified using 12% SDS–polyacrylamide gel electrophoresis (PAGE) followed by staining with Coomassie Blue R-250. A PD-10 desalting column was used to desalt the purified AK2 then the final AK2 concentration was determined using a Bradford assay.

### ELISA

Binding activities of AK2 in culture supernatant or of purified AK2 to rhCDK4 were assessed using non-competitive ELISA. First, microtiter plates were coated with of rhCDK4 protein (10 μg/mL, 100 μL per well) overnight at 4 ℃. After blocking of wells with 3% non-fat milk in PBST (PBS with 0.05% Tween-20), 100 μL of supernatant of *E. coli* AK2/HB2151 cell culture or 100 μl of purified AK2 (diluted to 10 μg/mL in 3% non-fat milk in PBST) was added to wells then plates were incubated for 2 h then washed three times with PBST (as for each subsequent incubation). Next, anti-V5 tag antibody and HRP-conjugated IgG were incubated sequentially (separated by PBST washes) then the final reaction was initiated by addition of an HRP-based colourimetric developing solution (51.4 mM Na_2_HPO_4_, 24.3 mM citric acid, 1 mg/mL o-phenylenediamine, 0.03% H_2_O_2_). Results for each well were obtained using a microplate reader (Thermo Labsystems) based on absorbance at 492 nm. The negative control for the IPTG-induced *E. coli* AK2/HB2151 culture supernatant was supernatant obtained without IPTG induction. Due to the absence of AK2 in PBST containing 3% non-fat milk, PBST with 3% non-fat milk served as the negative control for purified AK2, while wells coated with recombinant human prolyl hydroxylase 2 (rhPHD2) [27], recombinant human cyclooxygenase-2 (rhCOX-2) [46] or bovine serum albumin (BSA) served as irrelevant protein controls for rhCDK4.

For competitive ELISA detection of different antigens, overnight coating of 96-well microtiter plates (Nunc) with CDK4 (10 μg/mL) were conducted at 4 °C. After blocking of wells with PBST containing 3% non-fat milk, a pre-incubation mixture containing 50 μL AK2 (10 μg/mL in PBST with 3% non-fat milk) and 50 μL of solutions containing different concentrations of CDK4 or other proteins (5.0, 10.0, 15.0, 20.0, 40.0, 60.0, 80.0 μg/mL in PBST with 3% non-fat milk) were added to wells, with only AK2 added to wells of the negative control (NC) group. Next, wells were sequentially incubated with anti-V5 tag antibody and HRP-conjugated IgG (separated by PBST washes) then wells were developed by the addition of the abovementioned colourimetric developing solution and absorbances were read using a microplate reader (Thermo Labsystems) at a wavelength of 492 nm.

For competitive ELISA-based detection of different antibodies, wells of 96-well microtiter plates (Nunc) were coated with CDK4 (10 μg/mL) overnight at 4 °C. After blocking, wells were pre-incubated with 100 μL AK2 (10 μg/mL in PBST with 3% non-fat milk) for the experimental group, AD5 [34] (10 μg/mL in PBST with 3% non-fat milk) as an irrelevant scFv control, anti-CDK4 mouse monoclonal antibody (10 μg/mL in PBST with 3% non-fat milk) as the positive control or PBST with 3% non-fat milk as NC. After pre-incubation, anti-CDK4 rabbit polyclonal antibody was added to detect CDK4. After a 2-h incubation at 37 °C, the plate was washed with PBST followed by the addition of HRP-conjugated goat anti-rabbit antibody. The reaction was developed via addition of the abovementioned colourimetric developing solution then absorbances were measured using the abovementioned microplate reader.

The competitive inhibition rate was calculated using the following formula: Inhibition rate (%) = (OD_492_ of the negative control − OD_492_ of the test group) / OD_492_ of the negative control × 100%.

### Determination of AK2 binding affinity for rhCDK4

To determine the binding affinity of AK2 for rhCDK4 protein, non-competitive ELISAs were conducted as previously reported [32, 34, 35]. Briefly, the microtiter plates were coated with serially diluted rhCDK4 (8.0, 4.0, 2.0, 1.0 μg/mL) protein overnight at 4˚C then wells were washed with PBST. After blocking, serial dilutions of AK2 (8.000, 4.000, 2.000, 1.000, 0.500, 0.250, 0.125 μg/mL) were added to wells followed by incubation, with the same sequential experimental procedure used here as was used for the non-competitive ELISA. All measurements for each CDK4 and AK2 concentration were conducted in triplicate. To determine the correlation between the amount of AK2 (Ab′ or Ab) bound to CDK4 (Ag′ or Ag) per well and absorbance measurements at 492 nm, the following formula was used to calculate the affinity constant (*K*_aff_) for AK2: *K*_aff_ = (n − 1) / (n [Ab′] − [Ab]), n = [Ag] / [Ag′], where [Ab], and [Ab′] refer to the concentration of antibody related to half the maximum absorbance at a wavelength of 492 nm (OD‐100) calculated using a nonlinear regression approach for wells coated with [Ag] and [Ag′], respectively.

### Cell culture

Cultures of HeLa cells (cervical cancer cells) and MCF-7 cells (breast cancer cells) obtained from ATCC were maintained in DMEM complete medium with 10% fetal calf serum in an incubator at 37 °C with humidification and 5% CO_2_. Adherent cells were grown in culture flasks and every 3–5 days cultures were digested by trypsin to detach cells from the walls of flasks then cells were subcultured in new flasks.

### Western blot assay

Whole-cell lysates of HeLa cells and MCF-7 cells were obtained using a modified NP-40 lysis buffer-based method then were subjected to a 12% SDS-PAGE and electroblotted to transfer proteins to PVDF (polyvinylidene difluoride) membranes. Membranes were blocked at room temperature to prevent non-specific binding of antibody probes to membranes by immersion of membranes in PBST containing 5% non-fat milk for one hour. For identification of AK2, the membrane was incubated overnight at 4 °C with anti-His tag or anti-V5 tag antibody in PBST containing 5% non-fat milk using the manufacturer’s recommended dilution. For detection of AK2 binding to endogenous CDK4, the membrane was first incubated with AK2 at room temperature for two hours then was washed 3 times with PBST followed by an overnight incubation at 4 °C with anti-V5 tag antibody in PBST with 5% non-fat milk using the manufacturer’s recommended dilution. After washing the membrane 3 times with PBST, it was incubated with HRP-conjugated IgG at a 1:5000 dilution in PBST containing 5% non-fat milk for one hour at room temperature then was washed 3 times with PBST. Membranes were developed using a western blot detection system to reveal protein bands.

### Immunofluorescence assay

HeLa or MCF-7 cells were grown on sterile glass coverslips in 6-well culture plates. After 48 h of incubation, coverslips with cells were removed from the 6-well plates and immersed in 4% paraformaldehyde for 30 min to fix the cells. Next, fixed cells on coverslips were washed with PBS containing 0.1% Triton X-100 then cells were permeabilised by digestion with 0.01% trypsin. Next, coverslips were blocked by immersion in PBS containing 0.1% Triton X-100 and 3% BSA for 30 min. Coverslips with cells were sequentially incubated with AK2 (10 μg/mL in PBST with 0.1% Triton X-100 and 3% BSA) overnight at 4 °C then were washed and incubated with anti-V5 tag antibody for 2 h at room temperature. Coverslips were then washed and incubated with FITC-labelled rabbit anti-mouse IgG for 20 min at room temperature in the dark. Finally, Hoechst 33,342 dye was added at a final concentration of 2 μg/mL and coverslips with cells were incubated for 20 min in the dark. Each coverslip was mounted onto the stage of a fluorescence microscope and a drop of PBS containing 90% glycerol was applied to the upper surface of the coverslip then cells on coverslips were observed using excitation wavelengths of 350 nm and 460 nm.

### Co-immunoprecipitation (Co-IP)

HeLa or MCF-7 cells in 6-well culture plates were washed twice with ice-cold PBS containing 1 mM PMSF and lysed by addition to each well of 500 μL of cell lysis buffer (100 mM NaCl, 50 mM Tris, 0.25% NP‐40, protease inhibitor cocktail, pH 8.0). Adherent cells were scraped off the plate and lysed then cell lysates were transferred to microcentrifuge tubes followed by centrifugation of tubes at 15,000 rpm for 20 min at 4 °C. Supernatants were transferred to fresh tubes then were incubated sequentially under gentle rotation at 4 °C with purified AK2 for 2 h then with anti-V5 tag antibody for 2 h. Next, 30 μL of Protein A/G conjugated to Sepharose® 4B fast flow beads was added into each tube and tubes were slowly inverted and left overnight at 4 °C. The next day the tubes were centrifuged at 8000 rpm for 3 min at 4 °C and supernatants were discarded. After three times of washing with cell lysis buffer and cold PBS, the beads were treated with 25 μL of 2 × SDS loading buffer then subjected to SDS-PAGE followed by western blot analysis.

### Statistical analysis

All samples were tested in triplicate, with reported values representative of three independent experiments. All values were expressed as the mean ± standard deviation (SD) of three parallel measurements. Student’s t-test was adopted for data analysis and all test results were considered statistically significant at *P* < 0.05.

## Supplementary Information

**Additional file 1. Figure S1.** The amino acid sequences of AK2-VL and AK2-VH. VL: light chain variable region; VH: heavy chain variable region; CDR: complementarity determining region. **Figure S2.** The original blot image of Figure 2b. Western blot analysis of purified AK2 using anti-V5 tag monoclonal antibody and anti-His tag monoclonal antibody probes, respectively. Lanes 1 and 2: Cell lysate of *E. coli* AK2/HB2151 induced without or with IPTG, respectively; Lane 3: Culture supernatant of *E. coli* AK2/HB2151 induced by IPTG; Lane 4: Purified AK2 protein. Each blot showed a developed image along with a merge image of the developed photo and the white field photo. All cropped blot image parts in the manuscript are highlighted with red frames on the developed images. **Figure S3.** The original blot image of Figure 6a. Detection of binding between AK2 and CDK4 within cells as shown using western blot analysis. Proteins of HeLa and MCF-7 cells were separated using 12% SDS-PAGE then were transferred to PVDF membranes. PVDF membranes were sequentially probed with AK2 and anti-V5 tag antibody to detect CDK4, while PVDF membranes probed only with anti-V5 tag antibody served as the NC, while β-actin served as loading control.Each blot showed a developed image along with a mergeimage of the developed photo and the white field photo. All cropped blot images in the manuscript are highlighted with red frames on the developed images. **Figure S4**. The original blot image of Figure 6b. Analysis of interactions between AK2 and CDK4 in cells using co-immunoprecipitation assays. HeLa and MCF-7 cell lysates pre-incubated with AK2 were co-immunoprecipitated with anti-V5 tag antibody then were subjected to western blot analysis to detect CDK4 binding. Lysates without added purified AK2 served as the negative control (NC). Mouse IgG served as NC for probing with anti-V5 tag antibody. Western blots of HeLa and MCF-7 cell lysates served as input control, while β-tubulin served as loading control. Each blot showed a developed image along with a merge image of the developed photo and the white field photo. All cropped blot image parts in the manuscript are highlighted with red frames on the developed images.

## Data Availability

All authors declare that the data generated or analysed during this study are included in this published article and its supplementary information files.

## Abbreviations

CDK4: Cyclin-dependent kinase 4
scFv: Single-chain variable fragment
*E. **coli*: *Escherichia coli*
ELISA: Enzyme linked immunosorbent assay
Rb protein: Retinoblastoma protein
IPTG: Isopropyl β-D-1-thiogalactopyranoside
OPD: O-phenylenediamine
PBS: Phosphate buffered saline
PBST: PBS containing Tween-20
PHD2: Prolyl hydroxylase 2
COX-2: Cyclooxygenase-2
DMEM: Dulbecco's modified eagle medium
PVDF: Polyvinylidene difluoride
IF: Immunofluorescence
IP: Immunoprecipitation
BSA: Bovine serum albumin
