# Supplementary material for: Expression, purification and characterisation of a human anti-CDK4 single-chain variable fragment antibody
Source: BMC Biotechnol. 2021 Dec 20;21:71. doi: 10.1186/s12896-021-00729-z (PMC8690526; doi:10.1186/s12896-021-00729-z)
Supplement: Supplementary file 1 — Additional file 1. Figure S1. The amino acid sequences of AK2-VL and AK2-VH. VL: light chain variable region; VH: heavy chain variable region; CDR: complementarity determining region. Figure S2. The original blot image of Figure 2b. Western blot analysis of purified AK2 using anti-V5 tag monoclonal antibody and anti-His tag monoclonal antibody probes, respectively. Lanes 1 and 2: Cell lysate of E. coli AK2/HB2151 induced without or with IPTG, respectively; Lane 3: Culture supernatant of E. coli AK2/HB2151 induced by IPTG; Lane 4: Purified AK2 protein. Each blot showed a developed image along with a merge image of the developed photo and the white field photo. All cropped blot image parts in the manuscript are highlighted with red frames on the developed images. Figure S3. The original blot image of Figure 6a. Detection of binding between AK2 and CDK4 within cells as shown using western blot analysis. Proteins of HeLa and MCF-7 cells were separated using 12% SDS-PAGE then were transferred to PVDF membranes. PVDF membranes were sequentially probed with AK2 and anti-V5 tag antibody to detect CDK4, while PVDF membranes probed only with anti-V5 tag antibody served as the NC, while β-actin served as loading control.Each blot showed a developed image along with a mergeimage of the developed photo and the white field photo. All cropped blot images in the manuscript are highlighted with red frames on the developed images. Figure S4. The original blot image of Figure 6b. Analysis of interactions between AK2 and CDK4 in cells using co-immunoprecipitation assays. HeLa and MCF-7 cell lysates pre-incubated with AK2 were co-immunoprecipitated with anti-V5 tag antibody then were subjected to western blot analysis to detect CDK4 binding. Lysates without added purified AK2 served as the negative control (NC). Mouse IgG served as NC for probing with anti-V5 tag antibody. Western blots of HeLa and MCF-7 cell lysates served as input control, while β-tubulin served as loading control. Ea [file 12896_2021_729_MOESM1_ESM.pdf]

Expression, purification and characterisation of a human anti-CDK4 single-chain variable fragment antibody

Jialiang Zhao, Jingjing Xu, Tianbin Yang, Xinze Yu, Cheng Cheng, Tong Zhang, Ze Ren, Na Li, Fang Yang*, Guiying Li*

Key Laboratory for Molecular Enzymology and Engineering of the Ministry of Education, School of Life Sciences, Jilin University, Changchun 130012, China

Correspondence: Fang Yang, E-mail: fangyang@jlu.edu.cn;

Guiying Li, E-mail: ligy@jlu.edu.cn.

Supplementary figure legends

Figure S1. The amino acid sequences of AK2-VL and AK2-VH. VL:light chain variable region; VH: heavy chain variable region; CDR: complementarity determining region.

Figure S2. The original blot image of Figure 2b. Western blot analysis of purified AK2 using anti-V5 tag monoclonal antibody and anti-His tag monoclonal antibody probes, respectively. Lanes 1 and 2: Cell lysate of *E. coli* AK2/HB2151 induced without or with IPTG, respectively; Lane 3: Culture supernatant of *E. coli* AK2/HB2151 induced by IPTG; Lane 4: Purified AK2 protein. Each blot showed a developed image along with a merge image of the developed photo and the white field photo. All cropped blot image parts in the manuscript are highlighted with red frames on the developed images.

Figure S3. The original blot image of Figure 6a. Detection of binding between AK2 and CDK4 within cells as shown using western blot analysis. Proteins of HeLa and MCF-7 cells were separated using 12% SDS-PAGE then were transferred to PVDF membranes. PVDF membranes were sequentially probed with AK2 and anti-V5 tag antibody to detect CDK4, while PVDF membranes probed only with anti-V5 tag antibody served as the NC, while β-actin served as loading control.Each blot showed a developed image along with a merge image of the developed photo and the white field photo. All cropped blot images in the manuscript are highlighted with red frames on the developed images.

Figure S4. The original blot image of Figure 6b. Analysis of interactions between AK2 and CDK4 in cells using co-immunoprecipitation assays. HeLa and MCF-7 cell lysates pre-incubated with AK2 were co-immunoprecipitated with anti-V5 tag antibody then were subjected to western blot analysis to detect CDK4 binding. Lysates without added purified AK2 served as the negative control (NC). Mouse IgG served as NC for probing with anti-V5 tag antibody. Western blots of HeLa and MCF-7 cell lysates served as input control, while β-tubulin served as loading control. Each blot showed a developed image along with a merge image of the developed photo and the white field photo. All cropped blot image parts in the manuscript are highlighted with red frames on the developed images.

Figure S1

AK2-VL: QSALTQPPSASGSPGQSVTISCTGTSSDVGGYNYVSWYQQ 40

CDR1

HPGKAPKLMIYDVSKRPSGVSNRFSGSKSGNTASLTIFGL 80

CDR2

QAEDEADYYCSSYTSSSTLVFGGGTKLTVLG 111

CDR3

AK2-VH: TQVQLQESGGGLVQPGGSLRLSCAASGFTFSSYAMSWVRQ 40

CDR1

APGKGLEWVSAISGSGGSTYYADSVEGRFTISRDNSKNTL 80

CDR2

YLQMNSLRAEDTAVYYCARRLTGFDYWGQGTLVTVSS 117

CDR3

Figure S2

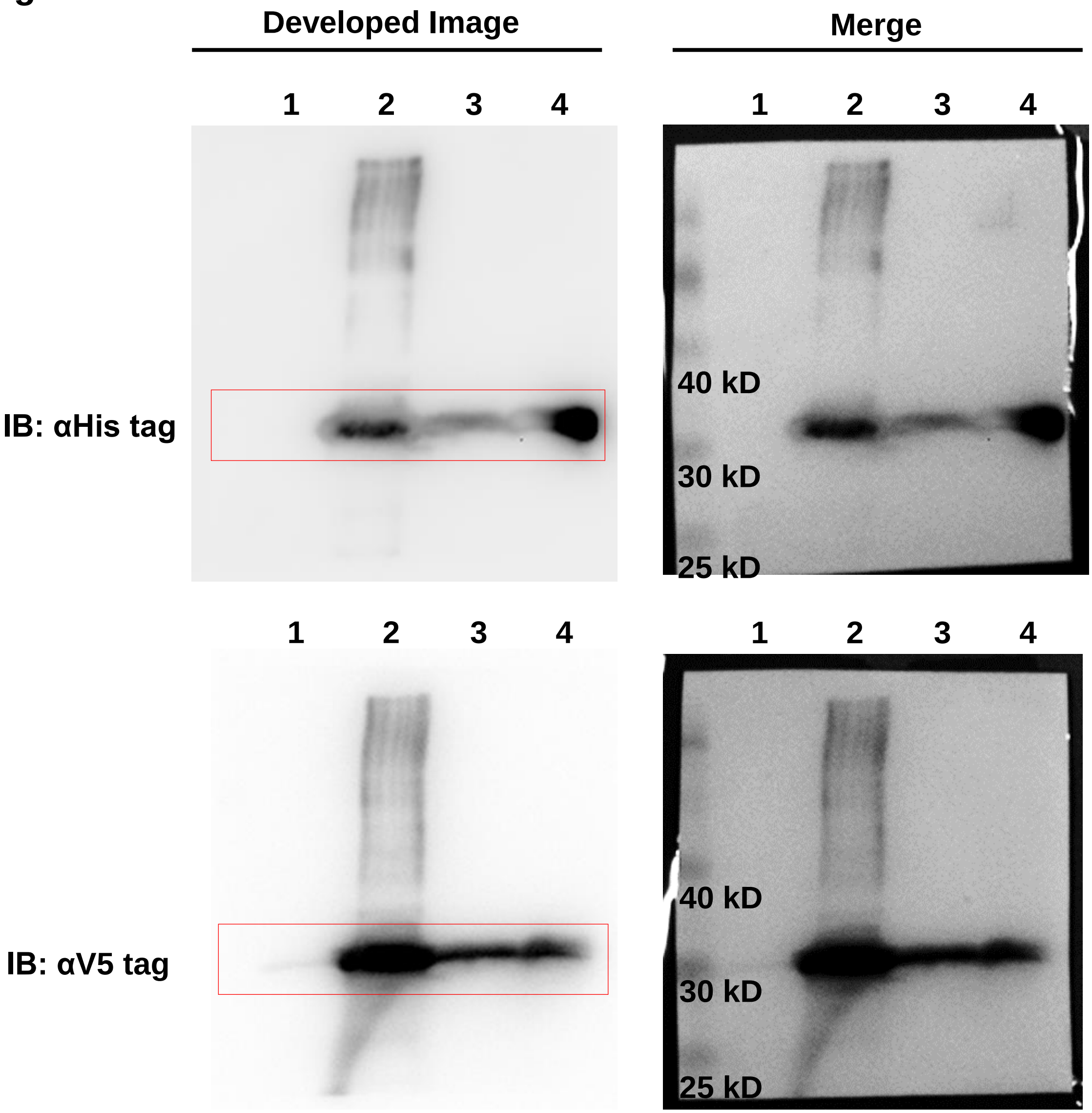

Developed Image
Merge
1
2
3
4
IB: αHis tag
40 kD
30 kD
25 kD
IB: αV5 tag

Figure S3

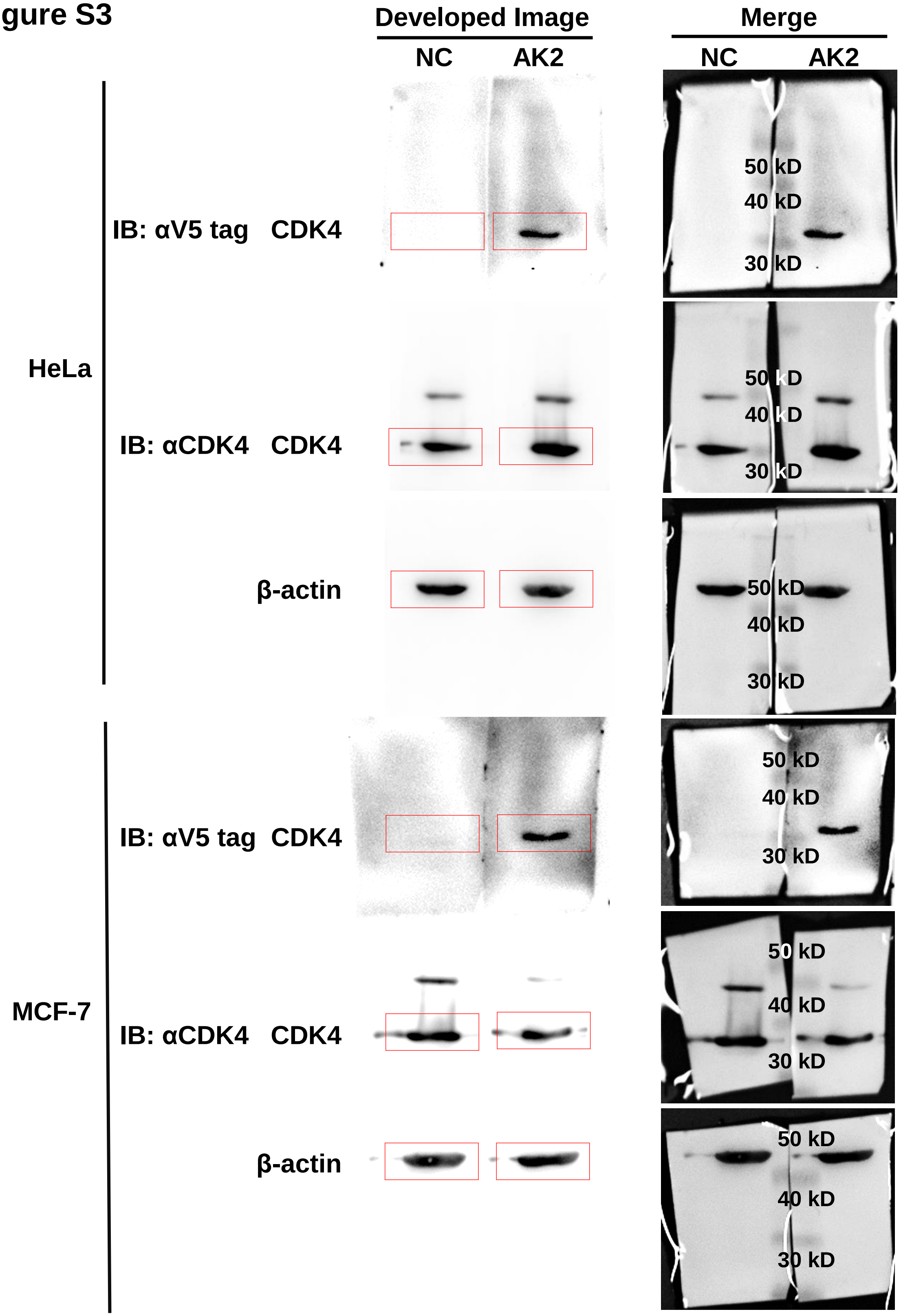

Developed Image
Merge
NC
AK2
NC
AK2
HeLa
IB: αV5 tag CDK4
IB: αCDK4 CDK4
β-actin
MCF-7
IB: αV5 tag CDK4
IB: αCDK4 CDK4
β-actin
50 kD
40 kD
30 kD

Figure S4

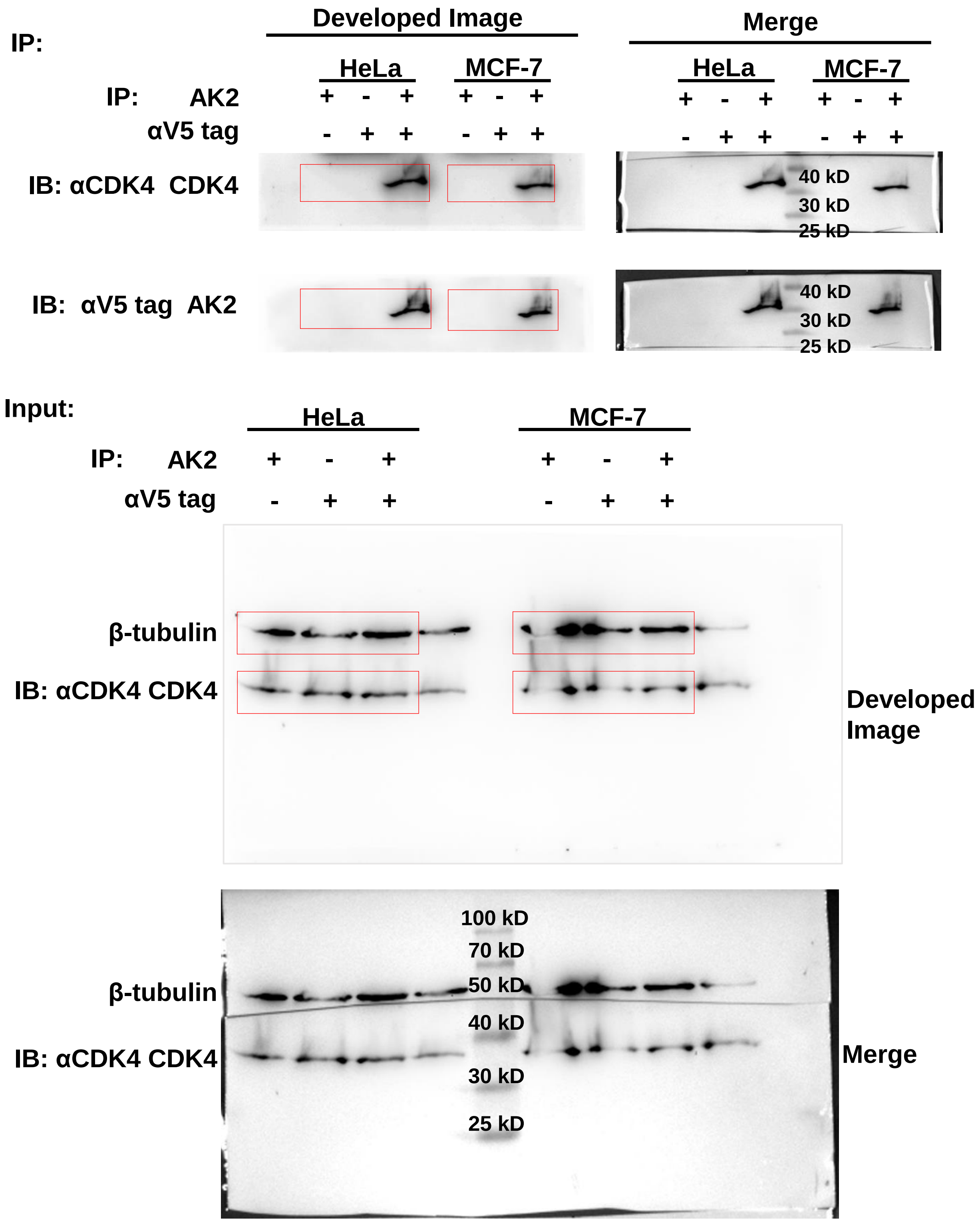
IP:
Developed Image
Merge
HeLa
MCF-7
IP: AK2
αV5 tag
IB: αCDK4 CDK4
IB: αV5 tag AK2
40 kD
30 kD
25 kD
Input:
β-tubulin
Developed Image
100 kD
70 kD
50 kD
Merge
